# Evolution of Experimental Models in the Study of Glioblastoma: Toward Finding Efficient Treatments

**DOI:** 10.3389/fonc.2020.614295

**Published:** 2021-01-29

**Authors:** Ricardo Gómez-Oliva, Samuel Domínguez-García, Livia Carrascal, Jessica Abalos-Martínez, Ricardo Pardillo-Díaz, Cristina Verástegui, Carmen Castro, Pedro Nunez-Abades, Noelia Geribaldi-Doldán

**Affiliations:** ^1^ Área de Fisiología, Facultad de Medicina, Universidad de Cádiz, Cádiz, Spain; ^2^ Instituto de Investigación e Innovación Biomédica de Cádiz (INIBICA), Cádiz, Spain; ^3^ Departamento de Fisiología, Facultad de Farmacia, Universidad de Sevilla, Sevilla, Spain; ^4^ Departamento de Anatomía y Embriología Humanas, Facultad de Medicina, Universidad de Cádiz, Cádiz, Spain

**Keywords:** glioma stem cells, cell cultures of glioma cells, mouse models of glioblastoma, brain organoids, 3D bioprinting

## Abstract

Glioblastoma (GBM) is the most common form of brain tumor characterized by its resistance to conventional therapies, including temozolomide, the most widely used chemotherapeutic agent in the treatment of GBM. Within the tumor, the presence of glioma stem cells (GSC) seems to be the reason for drug resistance. The discovery of GSC has boosted the search for new experimental models to study GBM, which allow the development of new GBM treatments targeting these cells. In here, we describe different strategies currently in use to study GBM. Initial GBM investigations were focused in the development of xenograft assays. Thereafter, techniques advanced to dissociate tumor cells into single-cell suspensions, which generate aggregates referred to as neurospheres, thus facilitating their selective expansion. Concomitantly, the finding of genes involved in the initiation and progression of GBM tumors, led to the generation of mice models for the GBM. The latest advances have been the use of GBM organoids or 3D-bioprinted mini-brains. 3D bio-printing mimics tissue cytoarchitecture by combining different types of cells interacting with each other and with extracellular matrix components. These *in vivo* models faithfully replicate human diseases in which the effect of new drugs can easily be tested. Based on recent data from human glioblastoma, this review critically evaluates the different experimental models used in the study of GB, including cell cultures, mouse models, brain organoids, and 3D bioprinting focusing in the advantages and disadvantages of each approach to understand the mechanisms involved in the progression and treatment response of this devastating disease.

## Introduction

Glioblastoma (GBM) is the most common and aggressive tumor of the central nervous system (CNS) (1). It represents more than 60% of all brain tumors in adults and it is associated with a bad prognosis. Also, the Central Brain Tumor Registry of the United State (CBTRUS) registered in 2019 (including dates ranged between 2012 and 2016) that GBM represents the 14.6% of all malignant brain tumors being male population the most affected (1.5 times more than female) by this disease (2, 3). Despite the efforts of scientists and clinicians to increase the life expectancy of GBM patients, survivors do not easily exceed the 15^th^ month (3–5) and the 5-year survival rate is as low as 5.8% (2, 3, 6). Furthermore, the median age is 65, and the most affected age ranges from 75 to 84 (3, 6). Globally, the incidence of GBM is higher in some specific areas over others, such as North America, the west and north of Europe, and Australia (7). Several risk factors have been studied as critical for GBM development such as constitutive genetic factors, ionizing radiation, or reduced susceptibility to suffer allergies and asthma. However, some inconsistencies among the different studies reveal the need of further investigations (8–11). These inconsistencies are likely to be caused by the existence of different types of GBM, which behave differently.

The World Health Organization (WHO) classified GBM in 2016 combining histopathological and molecular features (12). GBM are now classified into three subtypes based on the presence and absence of IDH mutations: GBM IDH-wild type (90% of tumors), IDH-mutant (10%), and GBM IDH-NOS in which a full IDH evaluation cannot be performed. GBM-IDH-wt include three variants: giant cell glioblastoma, gliosarcoma, and a novel and provisional variant, the epithelioid GBM characterized by large epithelioid cells and the presence of the *BRAF* V600E mutation (12, 13). Based on the expressions of genes, GBM have been classified in Classical, Mesenchymal, Proneural, and Neural (14). Classical GBM is characterized by an amplification of the chromosome 7, the loss of chromosome 10, and an increase in EGFR expression. In Mesenchymal subtype a focal deletion of *FN1 gene* is observed affecting the AKT pathway, whereas the NF-κB pathway is highly expressed. The Proneural subtype is characterized by alterations of *PDGFRA* and point mutations in IDH. In this subtype some genes such as SOX, DCX, ASCL1 are affected. The Neural type is characterized by the presence of neural markers such as *NEFL* or *GABRA1* (14). Several genetic alterations in GBM have been linked with recurrence and relapse. Thus, recurrent glioblastoma shows a higher frequency of copy number variations in several genes, particularly cell cycle genes, an enrichment in the cyclin-dependent kinase inhibitor 2A/B (CDKN2A/B) loss, and an excessive activation of cell cycle pathway genes. Also, gen sets such as TERT promoter and IDH1 mutation or tumor protein 53 (TP53) and IDH1 mutation (15).

Given the bad prognosis associated to this type of tumors, the search for therapeutic tools that represent a real increase in the survival rate has become the main goal in GBM research. Current GBM treatment includes the complete surgical resection of the tumor mass, followed by a combination of radiotherapy and chemotherapy (16). In this context, it is reasonable to say that the most significant development in clinical management of glioblastoma over the past two decades has been the groundbreaking trial of combining radiotherapy plus temozolomide (TMZ) (17), which resulted in an increase in the 2-year survival from 8% in patients with radiotherapy alone to 20% in patients with the combined therapy. Despite this improvement, effectiveness of treatment is variable from patient to patient. Apparently, effectiveness of treatment depends on several factors such as the tumor localization and size, or the brain anatomical structures affected (18). Essentially, one of the most relevant problems surrounding GBM is its infiltration into the healthy brain tissue, which makes practically impossible to perform a complete resection using surgical tools. In addition, the posterior radiation and chemotherapy do not completely eliminate all GBM cells (19). Thus, new insights in surgical tools are being used to allow visualization of cells within the tumor and improve the tumor mass resection. These are fluorescence-guided microsurgery (20) or intraoperative MRI, and ultrasound, which have been used in the surgical resection of CNS gliomas with the goal of maximizing extent of resection to improve patient outcomes (21). Regarding chemotherapy, TMZ is still the most effective so far, however, several other chemotherapeutic agents are being used, some of them directed to modulate the activation and suppression of signaling pathways altered in GBM. Examples of these new treatments are nelfinavir, tipifarnib, tamoxifen, or enzastaurin (22). These agents have proven not to be the most effective in individualized treatments, nonetheless, considering the molecular, cellular, histological, and genetic variances found in GBM, a deep molecular characterization of the different tumors could potentially allow the design of individualized therapies using these agents.

A problem linked to the inefficacy of TMZ treatment in the long term is that some cells within the tumor have the ability to escape its action (23) as well as the complementary radiation (24). These are the glioma stem cells (GSC). These cells share many similarities with Neural Stem Cell (NSCs) present within the physiological neurogenic niche of the subventricular zone (SVZ). These similarities are principally self-renewal and differentiation capacity (25, 26) in addition to several neurogenic markers, such as CD133 (27), nestin (28, 29), CD15 (30, 31), or some transcriptional factors including Sox2, Olig2, Nanog, and c-Myc (32). The first evidences on the role of the SVZ harboring malignant GBM cells were obtained using fluorescence guided resection of GBM using the commonly used fluorescent marker 5-aminolevulinic acid (5-ALA). These resections reveal the presence of fluorescent cells not only within the tumor mass but also in the adjacent SVZ, thus suggesting the presence of malignant cells in the SVZ of GBM patients (33). Clonal analysis of the stem cell populations suggested a GBM evolution as a result of multiple, genetically diverse clonal and sub-clonal populations involving both the SVZ and the tumor mass (33, 34). The role of the SVZ as a place of origin of GBM has gained strength because of the similarities between GSC and NSC of the SVZ (35). Vasculature, hypoxia, and several growth factors that promote GSC proliferation have been deeply studied in order to clarify the role of the SVZ in the origin of GBM (36–38). The human SVZ is characterized by presenting a complex cytoarchitecture composed of layers that provide a good environment to proliferation and differentiation of NSC (39, 40). In 2018, Lee et al. showed, using single-cell sequencing, that astrocyte-like NSC in the healthy SVZ tissue of GBM patients hide GBM driver mutations, cells that are capable of migrating from the SVZ to lead the development of high-grade malignant gliomas in distant brain regions (35).

**Figure 1:**
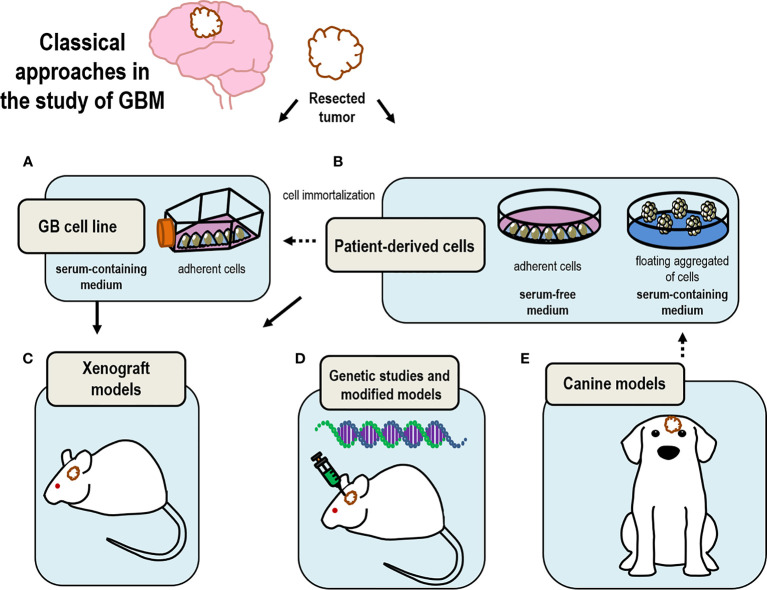
Drawing summary of classical models currently in use to study GBM. Tumor samples resected from patients upon surgery are used to **(A)** generate immortalized cell lines that can be maintained in time; **(B)** select GSC by culturing these cells as neurospheres under floating conditions; **(C)** produce xenograft models by transplanting either cultured isolated cells or tumor tissue; **(D)** perform genomic analysis to select candidate genes to produce genetically modified mice models of GBM; **(E)** canine models are good model for the study of GBM based on their analogies with human GBM.

**Figure 2:**
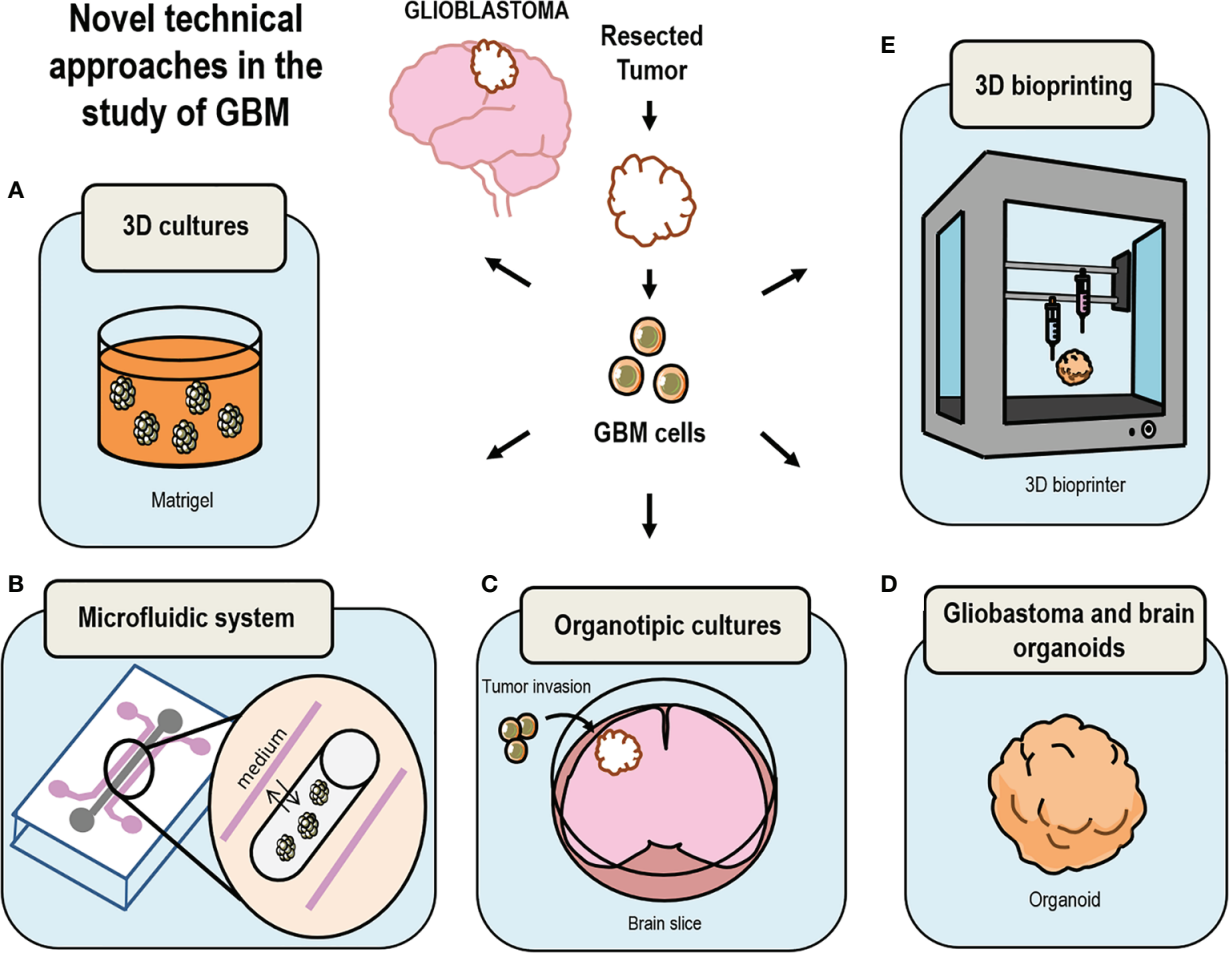
Drawing summary of novel models currently in use to study GBM. **(A)** 3D Cultures of cells embedded in Matrigel have been key techniques in the development of brain organoids; **(B)** growth of 3D structures using microfluidic systems replicates the changing microenvironment surrounding GBM in the human brain; **(C)** GBM cells can be grafted *in vitro* in human brain slices grown as organotypic cultures replicating the natural environment of a GBM tumor; **(D)** culture GBM organoids by either using glioma cells or by inserting GSC into brain organoids; **(E)** bio-print GBM 3D-spheroids using extracellular matrix materials and other cell types.

Over the past years a vast set of methodologies have been used in the study of GBM, leading to most of our current knowledge on tumor development and prognosis (41) (**Figure 1** and **Tables 1**, **2**). However, the past 10 years have been crucial in the development on new and innovative techniques, such as the growth of GBM organoids, which are leading to novel and individualized therapies (**Figure 2** and **Table 3**) for the treatment of this disorder (99, 127). In here, we discuss some classical methodologies together with the description of the most recently developed techniques to study GBM.

**Table 1:** Advantages and disadvantages of GBM experimental models.

Method of study	Advantage	Disadvantage	References
**GBM cell lines**	Good method to understand GBM biology Simple system provided with a single type of cells Less ethical concerns Reproducibility Fast method to obtain preliminary results Easy to manipulate	Does not allow studies about cell interactions or molecular mechanism in GBM development Does not represent GBM microenvironment Reduced genetic heterogeneity of cells compared with native tumor	Fields et al. (42), Sundarraj et al. (43), de Ridder et al. (44), Notarangelo et al. (45), Weeber et al. (46), Torsvik et al. (47), Allen et al. (48).
**Patient-derived cells**	Respect intratumor heterogenicity (GSC), tumor initiation, invasiveness process Genotype and phenotype characteristics resemble those of primary tumors Give more comparable results Can be maintained *in vitro*	Cell cultures are limited to 20–30 passages before cells start exhibiting genomic and transcriptional changes Quiescent GSC are deselected within the neurospheres upon the different passages Introduce errors in terms of clonality, size, and number of neurospheres	Sottoriva et al. (49), Jiang et al. (50), Linkous et al. (51), Lee et al. (52), Brewer et al. (53), Baskaran et al. (54), Jayakrishnan et al. (55), Wang et al. (56), Chen et al. (57), Mori et al. (58), Ladiwala et al. (59), Pollard et al. (60), Fael Al-Mayhani et al. (61), Rahman et al. (62).
**Xenograft model of GBM**	Allow personalized drug efficiency tests in single patients Maintain the original tumor architecture and histological characteristic Genetically stable	A nude mouse is necessary to develop this model Cells need to be cultured in spheroids forms before implantation Does not reproduce the original niche. It is not allow to test immunomodulatory therapies	Shu et al. (63), Lee et al. (52), Tentler et al. (64), Joo et al. (65), Patrizii et al. (66), Ashizawa et al. (67), Hutchinson et al. (68), Son et al. (69), Khaddoui et al. (70), Lynes et al. (71).
**Genetically engineered and viral vector-mediated transduction models**	Modified models offer the ability to directly alter the genome of somatic cells in mouse tissues introducing or removing specific genes Reproduce pre-clinical features Easy approach to rapidly analyze therapeutic responses to drugs	Genetics and histology of the modified tumor models are often not representative of the original human tumor	Furnari et al. (72), Holland et al. (73), Uhrbom et al. (74), Wei et al. (75), Baker et al. (76), Miyai et al. (77).
**Canine model of GBM**	Similarities with human glioma Good tool to perform pre-clinical studies	Ethical issues Difficult to detect GBM in canine models	Herranz et al. (78), Stoica et al. (79), Candolfi et al. (80), Chen et al. (81), Fernandez et al. (82).
**Organotypic cultures**	Good model to study invasiveness * Niche similarities	* Not reproduce interactions with blood flow factors or typical hypoxic conditions	Eisemann et al. (83), Sliwa et al. (84); Marques-Torrejon et al. (85), Ravi et al. (86)
**Brain organoids**	Provide a powerful tool for the *ex vivo* study of the molecular and cellular mechanisms Maintain the architecture and organization of tissues Preserve important features of the original tumor heterogeneity Useful to study GBM pathology and drug response Good method to design personalized therapies and treatments and, also, in drug screening	Difficult approach in terms of technology Need biopsies of patients Complex structures to maintain Elevated cost	Lancaster and Knoblich (87), Clevers (88), Chen et al. (89), Sasai (90), Huch et al. (91); Hubert et al. (92), da Silva et al. (93), Ogawa et al. (94), Bian et al. (95), Krieger et al. (96), Linkous et al. (51), Hwang et al. (97), Jacob et al. (98), Zhang et al. (99), Perrin et al. (100), Vlachogiannis et al. (101), Ooft et al. (102), Ganesh et al. (103), da Hora et al. (104).
**3D bioprinting**	Provide a cell network that resembles reality in a very faithful way Replicate the architecture of tissues Good method to test drugs effectively Good to analyze cell signaling Good method to test drug sensibility scanning Good method to study invasiveness process, immunologic interactions and cellular crosstalk	Need photo-crosslinking Need effective biomaterials, which do not affect normal tissue development Printing resolution still needs to be improved Need of a bioprinter High cost	Roseti et al. (105), Heinrich et al. (106), Ananthanarayanan et al. (107), Xiao et al. (108), Hermida et al. (109), Tang et al. (110)

**Table 2:** Current trends in mouse xenografts and allografts.

Reference	Implantation type	Cell type	Tumor type	Animal model	Strategy used
Tateishi et al. (111)	Orthotopic xenograft	MGG152	Recurrent GBM	SCID mice	NAMPT inhibitor
		HT1080	Fibrosarcoma		
Ashizawa et al. (67)	Heterotopic xenograft	GB-SCC010	Primary GBM	NOD-SCID mice and NOG mice	STAT3 inhibitor
		GB-SCC026	Primary GBM		
Szabo et al. (112)	Orthotopic xenograft	LNT-229	GBM	Nude mice	Neutralization with VEGF or PlGF antibody
		LN-308	GBM		
Sharpe et al. (113)	Orthotopic xenograft	BT111	Primary GBM	Nude mice	Monoamine oxidase B-activated pro-drug
		BT116	Primary GBM		
Zhang et al. (114)	Orthotopic xenograft	LN-319	GBM	NSG mice	ErbB2/HER2-Specific NK Cells
	Orthotopic allograft	GL261	GBM		
Parrish et al. (115)	Heterotopic and orthotopic xenograft	GBM12	Primary GBM	Mdr1a/b^−/−^Bcrp1^−/−^ knockout and wild type mice	PARP inhibitors and temozolomide
Garros-Regulez et al. (116)	Heterotopic xenograft	U251	GBM	Nude mice	mTOR inhibition with Rapamycin and Temozolomide
Karpel-Massler et al. (117)	Orthotopic xenograft	GBM164	Primary GBM	SCID mice	Bcl-xL inhibition with ABT263
		U87MG	GBM		
Yuan et al. (118)	Orthotopic xenograft	Patient derived brain tumor initiating cells	Primary GBM	SCID mice	ABT-888 and temozolomide treatment
Chang et al. (119)	Orthotopic xenograft	LN229	GBM	Nude mice	Pyr3 treatment
		U87MG	GBM		
Sun et al. (120)	Orthotopic xenograft	TT150630	Primary GBM	B-NDG mice	Palbociclib in treatment
		TT150728	Primary GBM		
Bejarano et al. (121)	Heterotopic xenograft	h676	Primary GBM	Nude mice	TRF1 Chemical Inhibitors
		h543	Primary GBM		
Guo et al. (122)	Orthotopic allograft	GL261, GL261 Red-FLuc and GL261-Luc2	GBM	C57BL mice	FTY720 treatment
Gravina et al. (123)	Heterotopic and orthotopic xenograft	U87MG	GBM	Nude mice	RES529, a TORC1/TORC2 dissociative inhibitor
Zalles et al. (124)	Orthotopic xenograft	G55	GBM	Nude mice	Neutralization with ELTD1 antibody
Jensen et al. (125)	Orthotopic xenograft	patient-derived GB brain tumor stem cells	Primary GBM	SCID mice	Afatinib and pacritinib treatment
Yang et al. (126)	Heterotopic and orthotopic allograft	GL261	GBM	C57BL/6 mice	Bip inhibition and ionizing radiation

**Table 3:** Current preclinical trials using organoids for the study of GBM.

Reference	GBM organoid from	Contribution to GBM study	Potential clinical use	Advantage	Disadvantage
Hubert et al. (92)	Patient-derived GBSC	Microenvironmental impact and cancer stem cell biology	Tumor sensitivity to radio-chemotherapy	Preserve phenotypic heterogenicity and tumorigenic capacity	No vessels No immune cells Slow growth No interaction with no-tumor cell
Ogawa et al. (94)	hESC-derived cerebral organoid genetically modified by introducing HRas^G12V^ through CRISPR/Cas9	Model for tumor formation and transplantation	Platform to test human cancer phenotypes and personalized therapy	Interactions between tumor and non- tumor cell	No vessels No immune cells Slow growth
Bian et al. (95)	hESC/iPSC-derived cerebral organoid genetically modified by introducing several mutation combinations through CRISPR/Cas9	GBM invasiveness and evaluation of drug response	Personalized therapy	Interactions between tumor and non- tumor cell	No vessels No immune cells Slow growth
Da Silva et al. (93)	Co-culture of human GBM spheroids with mouse ESC	Identification of anti-GBM invasion strategies	Drug screening. Tumor sensitivity to radio-chemotherapy	Interactions between tumor and non- tumor cell	No vessels No immune cells
Linkous et al. (51)	Co-culture of patient derived GBM stem cells with hESC-derived cerebral organoid	GBM biology in the human brain environment.	Drug screening Tumor sensitivity to radio-chemotherapy	Interactions between tumor and non- tumor cell	No vessels No immune cells
Krieger et al. (96)	GBM cells co-cultured with hESC-organoid cells	Invasion and transcriptional heterogeneity	Drug screening Tumor sensitivity to radio-chemotherapy	Interactions between tumor and non- tumor cell	No vessels No immune cells
Jacob et al. (98)	Patient-derived resected glioblastoma tumor tissue	GBM cells heterogeneity, tumor microenvironment and GBM infiltration	Useful to test therapy response including immunotherapy Construction of a living organism bank Biobank	Fast growth Vessels and immune cells	No interaction with non-tumor cell
Hwang et al. (97)	Patient derived- IPSC with c-met mutation	Reproduction a genomic network described in the most aggressive primary human GBM	Therapy response for GBM with c-met mutation	Good model to study progression and aggressiveness	No vessels No immune cells Slow growth No interaction with non-tumor cell

## Classical Approaches in the Study of Glioblastoma

### Cell Cultures of Glioblastoma Cells

One of the most important tools in the study of GBM has been the use of cell cultures. Cell cultures provide the maintenance of cells *in vitro* for research and clinical studies, however it is important to choose appropriately the cell line and culture because none of the currently available cell-based glioma model systems is able to reproduce the complex microenvironment of glioma cells within the brain. Thus, each *in vitro* model has its advantages and disadvantages and it is necessary to select the cell system appropriate for each experimental question (128). Some of these systems have been widely used not only in the study of GSC but also in the study of NSC giving a great deal of information about the physiology of these cells (129–132) or about the finding of small pharmacological molecules that regulate these activities (133).

### Glioblastoma Cell Lines

The first studies using cell-based glioma model systems used cell lines derived from induced Wistar/Furth rats and C57BL/6 mouse tumors of the central and peripheral nervous system (42, 43). Later on, human GBM cells were also immortalized for its use in culture (44, 45). This allowed the better understanding of glioma cell biology by simplifying the studies, since glioma cell lines provided an unlimited supply of cells available without ethical concerns and the possibility of obtaining reproducible results. Most commonly used human GBM immortalized cell lines are U87MG, U252, T98G, and LN-229. These cell lines show enrichment of cancer stem cells when grown as spheres in serum containing medium (134). To date, it is the fastest way to obtain preliminary results regarding the test of new anti-tumor drugs *in vitro.* GBM cell lines are easy to manipulate and to maintain in cultures using serum-containing media. However, cell lines do not provide a reliable model to understand the cellular and molecular mechanism underlying the development of GBM or to evaluate therapeutic interactions because it fails to represent the native tumor microenvironment. In addition, the use of glioma cell cultures holds other issues intrinsic to immortalized cell lines. Successive cell passages select cells which have the highest proliferative potential, decreasing the genetic heterogeneity found in the parental tumor (46). It is likely that the selection imposed by the passages in *in vitro* cultures result in genetic drift, accumulation of chromosomal aberrations, and phenotypic alterations in cell lines (47, 48). Because of these drawbacks, biobanks containing annotated and validated cell lines derived from surgical samples of GBM patients that preserve GSC features are being developed. This strategy provides an open resource for the study of a large part of GBM diversity (135).

Usually, the use of cell lines is well received as an approximation or as preliminary data but it is necessary the use of other models closer to reality to obtain more relevant and representative data. However, these cultures have been fundamental to solve and understand GBM biology (**Figure 1A** and **Table 1**).

### Patient-Derived Cells

Most cancers, including GBM, display intratumor heterogeneity and this could be one of the reasons why some tumors lack of satisfactory treatment (49, 50). Thus, the use of primary cell cultures derived from patients may facilitate the individualized study of GBM. Evidences show that the GSC subpopulation of GBM are very important to maintain tumor heterogeneity, as well as, the tumor initiation, maintenance, and invasion *in vivo* because of their capacity of self-renewal and differentiation (51). So far, GSC cultures have become the most accepted standard for studying GBM biology *in vitro* (52).

One of the problems found when using patient derived cultures is the use of serum since the subpopulation of GSC within the GBM is not present in cell cultures after prolonged serum exposure. GSC differentiate under these conditions, losing many of primary tumor characteristics (52). To avoid this issue, patient-derived GSC can be maintained *in vitro* under floating conditions in a serum free medium in which they form characteristic aggregates referred to as neurospheres. The starting population of cells is usually plated as a single-cell suspension in a non-adhesive substrate containing defined media supplemented with fibroblast growth factor (bFGF) and/or epidermal growth factor (EGF) (52, 53). However, it is important to consider that useful life of patient-derived glioma cell cultures is limited. After 20–30 passages cells start exhibiting genomic and transcriptional changes in metabolic and signaling pathways such as ribosomal synthesis, telomere packaging, or Wnt signaling pathways among others (54). Interestingly, the success rates of neurosphere cultures from gliomas is dependent on tumor grade and genetics, being the low-grade glioma cell cultures the most difficult to culture as neurospheres (55).

Despite its advantages, the neurosphere system presents a few drawbacks inherent to this cell culture method. Quiescent GSC may be lost with time since along the successive cell passages cells with the highest proliferative potential are selected and the subpopulation of quiescent GSC is deselected. This subpopulation is particularly important because it is believed to be responsible for chemotherapy and radiotherapy resistance (56, 57). Moreover, to ensure clonality and multipotentiality of neurospheres, cells should be seeded as single cells per well (58). However, this leads to a low number of cells because the concentration of paracrine and cell-to-cell signals required for cell growth and division are minimal under these conditions. On the contrary, neurospheres obtained after plating multiple cells per well show spontaneous locomotion resulting in cell aggregation and producing errors in terms of clonality, size, and number of neurospheres, thus leading to discrepancies in results and conclusions (59).

Looking for an effective manner to cultivate GSC, some reports describe a methodology to attach these cells onto a surface to expand them in serum free medium as adherent 2D cultures in the presence of growth factors EGF and bFGF (60, 61). The use of adherent 2D-cultures increases the efficiency of culture expansion avoiding the differentiation and apoptosis associated to the sphere cultures. Attached cells are more exposed to growth factors used in cultures that maintain the proliferative capacity (60). Other authors maintain that both methods, sphere and adherent cultures, are equal and useful to study GBM (62) and it is likely that a combination of both models is the best strategy for the *in vitro* test of GBM drugs, or for the study of new specific markers of GBM malignancy and progression.

Taking together all these facts prove the benefits of using this neurosphere cultures, starting with the capacity to preserve heterogeneity and also, to study the migration process and initiation of a new tumor. All these considerations are not present in cell lines models. However, the problem associated with the loss of quiescent GSC could be unfavorable in some studies. Nonetheless, this model allows the culture of cells from single individual patients, facilitating the study of inter-individual differences (**Figure 1B** and **Table 1**).

### Xenograft Models of Glioblastoma

*In vivo* transplants of GBM in animal models have long been used to study tumor development upon the engraftment of human cells into immunodeficient mice. Transplants of patient derived xenografts, in which dissociated tumor cells or tumor tissue fragments are implanted into mouse brains have successfully been used in murine models (**Figure 1C**). Mainly, three types of mouse have been used, which are classified according to their immune response: i) nude mice which are unable to produce T cells; ii) non-obese diabetic severe combined immunodeficiency (NOD-SCID) and SCID-beige mice, which lack T and B cells; and iii) NOD-SCID IL2R-γ null (NSG or NOG) mice, which lack T, B, or NK cell activity (136). GBM implantation is usually done in the subcutaneous flank location (heterotopic implantation) facilitating the visual observation of tumor development, which allows testing the efficacy of anti-tumor drugs by analyzing the tumor dimensions. However, an important limitation to heterotopic models is that the established microenvironment has an important role in GBM tumors. For this reason, xenografts based on the implantation of GBM in the brain (orthotopic implantation) are more extensively used nowadays because it provides a CNS microenvironment, and preserves the integrity of tumor-initiating cells (63). We have summarized in **Table 2** series of studies involving PDX models, showing how orthotopic or heterotopic xenografts have been used in different immunodeficient mouse strains to perform preclinical studies on the efficacy of novel drug treatments.

Invasive xenografts have been established from surgical specimens that were first maintained as tissue spheroids in short-term culture to obtain cell lines (137, 138). Many human and mouse cell lines have been used in PDX models (111, 112, 139, 140). The patient-derived cells are engrafted into immunocompromised mice to maintain the histopathologic, genomic, and phenotypic characteristic of the primary tumor across early passages (64–66). Lee et al. used GSC lines to initiate tumors in mice and they found a high percentage of engraftments compared with patient derived tumor xenografts. This type of tumor induction was much more potent and maintained the heterogeneity typical of GBM (35, 67). While NSC implantation into nude mice does not result in tumor formation, GSC implantation leads to the generation of tumors in a healthy brain, preserving tumor heterogeneity. This strategy opens the possibility to development of personalized drugs facilitating individual drug-screening and the search for resistance mechanisms. Notwithstanding, several drugs showing favorable results in pre-clinical studies using PDX have failed posteriorly in clinical trials (68). Indeed, this is not completely surprising since orthotropic implantation in PDX models does not reproduce the conditions of the niche of origin. The human stroma and microenvironment are not similar to those in the mouse, limiting the study to tumor biology and therapy resistance (69). Moreover, since these mouse models lack inflammatory responsive cells or an intact immune system it does not allow testing for immunomodulatory therapies. This limitation is currently critical since immune therapeutics have recently been very successful in treating a diverse group of cancerous lesions and there has been an explosion in the study of immune therapeutics for cancer treatment over the past few years (70, 71).

Among the advantages of these xenograft models three can be highlighted: i) they allow personalized drug efficiency tests in single patients, ii) the original tumor architecture and histological characteristic are preserved and iii) they are genetically stable (**Tables 1** and **2**). However, there are important caveats that need to be addressed in xenograft models: i) immunodeficient rodents may not respond to certain drugs, ii) the surrounding microenvironment of mouse origin may interfere with drug response, and iii) they do not allow the test of immune therapies, thus limiting the type of drugs to be tested.

### Genetically Engineered and Viral Vector-Mediated Transduction Mouse Models

As mentioned before, several genetic alterations have been found after the analysis of large numbers of uncultured GBM tissue samples removed from patients, leading to the discovery of several genes commonly mutated in GBM. Some of these mutations are already present in common cancer genes, such as EGFR, BRAF, RAS, PIK3CA, PIK3R1, PTEN (141, 142), particularly, EGFR variant III (EGFRvIII) is the most common active EGFR mutant in GBM. The presence of this mutant correlates with a poor patient prognosis due to its ability to extend downstream signaling (143). Accordingly, kinases like v-Src, which regulate the activity of these type of receptors have also been involved proliferation and migration of glioma cells (144). Additionally, a very clinically relevant discovery was the finding of the IDH1 mutation appears in a high percentage of secondary GBM and a small percentage of primary GBM (142). While PTEN loss, and EGFR amplification, are associated with primary glioblastoma, IDH1 mutation is common in secondary glioblastoma and show a higher survival rate.

The study of genetic alterations has led to the development of genetically engineered mouse models of GBM (77). Transgenic mouse models and knockouts as well as vector-mediated genetic approaches accurately reproduce pre-clinical features of GBM including the accumulation of genetic and epigenetic alterations in tumor suppressor genes or to the activation of oncogenic pathways, which lead to the progression of tumors (72, 142, 145). There are several examples employed in the development of mouse glioma modelling of aberrant expression of relevant downstream signaling pathways, resulting from i) expressing v-src kinase under the control of glial fibrillary acidic protein (GFAP) gene regulatory elements (77, 146), ii) aberrantly activating the p21-RAS signaling pathway mimicking the effect of EFGRvIII mutation (147, 148) to iii) overexpression of IDH1^R132H^ in the SVZ of the adult mouse brain (149).

In addition transduction of genes with viral vectors have been used to efficiently reproduced GBM development: i) retroviruses engineered to express relevant gain-of-function genes that result from overexpression of the viral oncogene v-sis, the cellular counterpart of which is c-sis or PDGF-B (73, 74); ii) adenovirus containing the EGFRvIII mutant into mice harboring activated RAS, which led to the efficient formation of glioblastoma (75); or iii) lentivirus expressing oncogenes such as HRAS or AKT (150, 151).

Models used in the study of GBM are the knockout of *p53* tumor suppressor gene harboring a conditional allele of the tumor suppressor *Nf1* (152), a model that displays an upregulation of Ras signaling; the Cdkn2a knockout mice combined with *Kras* and *Akt* upregulation by viral induction (74); the combination of both p53 and Cdkn2a knockout; the introduction of EGFRvIII or *Pten* loss in the glioma-prone mouse strain RasB8 mice with activated HRAS; the combined conditional knockout of tumor suppressors *p53, Nf1* and *Pten* genes (151).

Genetically engineered or viral vector-mediated models offer the ability to directly modify the genome of somatic cells in mouse tissues (by targeting the genomic alterations driving tumor behavior) for a rapid generation of complex mouse tumor models, that harbor specific genetic alterations providing the chance to potentially study specific drugs interfering with the function of such genes (76, 77) (**Figure 1D** and **Table 1**).

### Canine Models

The use of canine models to understand GBM biology represents a good option to simulate human conditions. The incidence of brain tumors in dogs is high and its characteristics are similar to those found in human (78, 79). Also, the comparison of GBM histopathological features in dog *versus* those in murine models leads to the conclusion of dog models as a good tool to perform preclinical studies (80, 81). The advantages of using the canine models of glioma are based on the similarities with human gliomas including the presence of several neural precursor markers such as nestin (82) and the capacity to form spheres (78). In contrast, an intrinsic problem to its use is the difficulty of detecting canine brain tumors and the ethical issues involved and the need to comply with the 3R (refinement, replacement, and reduction) for animal use in experimentation (**Figure 1E** and **Table 1**).

## Novel Technical Approaches in the Study of Glioblastoma

As it can be inferred from the studies discussed above, *in vitro* cell culture systems for the study of GBM do not exactly reproduce the real conditions surrounding brain tumors and the use of animal models may not be the best approach either to reproduce the particular niche in which GBM cells reside inside a human brain. Thus, a refinement of the *in vitro* techniques was required to produce humanized GBM models based on the three-dimensional (3D) culture of GBM cells in a system that reproduced the microenvironment of human brain tumors (**Figure 2**).

### 2D vs 3D Co-Cultures

Studies on GBM require the culture of patient derived GBM reproducing *in vitro* the conditions that establish interactions between different types of cells and between the cells with the extracellular matrix. GBM include a combination of fibroblasts, endothelial cells, and stem cells that release signaling ligands that determinate the characteristic. One of the challenges in cell culture for GBM has been the search for coating elements that reproduce these conditions as opposed to the effect of plastic. In this context a collection of hydrogels have been produced from ECM derived polymers that replicate the GBM microenvironment such hyaluronic acid, chitosan, chondroitin sulfate polysaccharides, alginates, and collagen/gelatin proteins (153). Matrigel (a mix of mouse collagen, laminin, and ECM-associated growth factors) is a hydrogel widely used in cultures of GBM, which allows cells to grow interacting on multiple sides (154). However, these conditions do not faithfully reflect the situation *in vivo* since proper tissue architecture and cell-cell contacts may be lost in such 2D systems as well as the contacts with the extracellular matrix.

Thus, recent works aimed to produce 3D GBM structures by culturing GBM cells onto hydrogel coated 3D scaffolds in which GSC or pieces of patient derived GBM are cultured reproducing a more real tumor environment (**Figure 2A**). These systems reproduce cell growth environment of GBM cells in combination with more than one type of cell, soluble signaling factors as well as the extracellular matrix signaling. Matrigel-coated 3D polystyrene scaffolds have already been successfully used to test drug efficacy (155), morphological structures in human tumors (156) and invasion (reviewed in Caragher et al., 2019) (154). The limitations of these scaffolds are the substrate stiffness, the selection across passages of cells that attach more loosely to the scaffold, which in turn are more invasive and apparently show expression patterns more similar to GSC. Also, the mouse origin of Matrigel makes it different from human brain ECM and more humanized Matrigel is being developed (154) together with other new promising biomaterials (157).

### Microfluidic Technology in Glioblastoma

GBM tumors are structures surrounded by a constantly changing microenvironment, which leads its development, however, the particular invariable conditions of the medium in 3D cultures do not reproduce this important attribute. Microfluidic technologies solve the problem of GBM cells growing in static medium (**Figure 2B**). These systems allow liquid media to be continually delivered to growing cells (158, 159). GBM primary cells can be grown in a scaffold of hydrogel tubes with circulating medium. Cells are pumped into the tubes in a solution containing brain ECM elements (160). The latest advances in this field include several devices that allow the maintenance of tissue for 3–7 days facilitating drug tests. As an example Olubajo et al. fabricated a device using glass in a photolithographic process, getting a high percentage (61.1%) of tissue viability compared with fresh tissue at the beginning of the experiment (68.9%) (159). This approach constitutes a new way to study GBM progression since it replicates microenvironmental and extracellular conditions prevailing in the brain and facilitates the measurement of biological phenomena with high resolution and in a high-throughput manner. In this context, Ayuso et al. used a new microfluidic model in GBM to understand GBM aggressiveness related to blood vessel obstruction. They studied the area surrounding the necrotic part of the tumor usually known as pseudopalisades finding a new method to study nutrient and oxygen behavior in tumor progression and in the migratory cell response (161).

All of these findings and promising methods have several benefits such as the reproduction of the environmental conditions typical of GBM, the generation of specific targeted drug tests, and the possibility of keeping the tissue for longer periods of time. On the contrary, it entails economic and time cost and the search for the adequate materials to deliver the microfluids depending on the type of study.

### Organotypic Cultures

A new but not so novel approach in the study of GBM is the use of organotypic cultures (**Figure 2C** and **Table 1**). These systems widely used in other types of studies allow the transplantation of GBM cells into brain slices that are kept alive for several weeks. This type of organotypic cultures have already been used for the study of tumor cell invasion the role of microglia in tumor growth and the niche factors governing tumor growth (83–85). In addition, microinjection of patient-derived tumor cells into cultured sections the use of human slices allows the study of GBM progression in its natural environment (86). This method enables GBM cells to be grown surrounded by cells like those in the niche of origin and therefore it is a good system to test microenvironmental interaction, however, it does not reproduce the interactions with blood flow factors or the hypoxic conditions.

### Glioblastoma and Brain Organoids

Brain organoids are a promising new technology that has offered new perspective for disease modelling, including cancer, in human tissues (87, 162, 163). These brain like structures, so called “mini-brains” provide a powerful tool for the *ex vivo* study of the molecular and cellular mechanisms of human brain disorders as they can accurately represent human organ histology and physiology (87–89). Brain organoids involve the generation of 3D tissues from pluripotent stem cells, such as induced pluripotent stem cells and embryonic stem cells, or adult-tissue-resident cells, that, in a controlled environment, slowly grow and differentiate (**Figure 2D** and **Table 1**). This architecture arises from the great self-organizing ability of these cells to form whole tissues (90, 91). Brain organoids from different regions are constructed resembling their *in vivo* counterpart and recapitulating at least some functions found *in vivo*. Forebrain organoids exhibit the multi-layer progenitor zone organization that recapitulates human cortical development, including a prominent SVZ layer with radial glial cells-exclusive expression of defined molecular markers. Besides, these organoids present a diverse collection of functional neuronal and other cell types found in developing human brains. Midbrain and hypothalamic organoids from human pluripotent stem cells have also been developed showing specific neuronal markers found *in vivo* (164).

Based on this technology, several laboratories have attempted to developed GBM models using different approaches:

As a first approach GBM specimens were embedded in Matrigel and cultured in serum free conditions in the presence of growth factors EGF and bFGF. This pioneering study proved the suitability of this method to GBM studies (92). This organoid model of GBM preserved important features of the original tumor such as phenotypic heterogeneity among stem cells as well as a hypoxic gradient that regulated stem cell mitotic activity. Also, they successfully demonstrated its high tumorigenic capacity after implantation in mouse brain. As an alternative approach, da Silva et al. co-cultured human GBM spheroids with early-stage brain organoids forming a hybrid organoid with spontaneous infiltration of tumor cells into the organoid demonstrating and invasive tumor phenotype (93).

A recent study has genetically engineered brain organoids to generate a GBM model. Thus, Ogawa et al. generated a GBM model organoid by entering the HRasG12V oncogene into human brain organoids modifying the fourth exon of TP53 locus through CRISPR/Cas9. This mutated cell which profile resembles the aggressive mesenchymal subtype of GBM, proliferate and invade the normal organoid speedily. Besides, in this work they also demonstrated that primary human-patient-derived glioblastoma cell lines can be transplanted into human cerebral organoids to induce tumors (94). In a related study, Bian et al. introduce different oncogenic mutations found in GBM into human cerebral organoids to study GBM pathology and evaluate drug response (95).

Linkous et al. developed an organoid model by using patient-derived glioma stem cells and human embryonic stem cell. This model called GLICO showed that GSC move into the human brain organoid invading and proliferating within the host tissue and forming tumors that closely phenocopy patient GBMs (51). Additionally, Krieger et al., co-cultured GBM cells with hESC-organoid cells and showed that tumor cells within organoids extend a network of long microtubes, recapitulating the *in vivo* behavior of GBM. They also demonstrated that transcriptional changes implicated in the invasion process are coherent across patient samples, indicating that GBM cells reactively upregulate genes required for their dispersion (96). In a different approach, Hwang et al. generated a neuronal organoid model mimicking GBM using induced pluripotent stem cells from a patient with c-met mutation, a mutation in receptor for hepatocyte growth factor involved in the progression and aggressiveness of GBM (97).

Finally, a recent study by Jacob et al. generated and created a live biobank of GBM organoids, called GBO, from fresh tumor without single-cell dissociation that mimic inter- and intra-tumoral heterogeneity and key aspects of their corresponding original tumors. These GBOs can be successfully transplanted into the adult mouse brain with an aggressive and fast infiltration profile and preserving original mutation expression (98). The field of GBM organoids has rapidly developed years, we have summarized in **Table 3** how this type of studies have progressed along the past four years from the first study by Hubert et al. (92) to the more recent advances.

In general, this revolutionary and developing technology reviewed by Zhang et al. (99) and summarize in **Table 3**, provided its limitations such as the lack of vasculature, immune cells or blood-brain barrier functions (100) has many advantages in GBM studies because it allows to: i) analyze the interactions between tumor and non-tumor cells (94, 95), ii) functionally analyze the consequences of genome aberrations within the same genetic background (94, 95), iii) study the interactions between tumor cells and their microenvironment, iv) test the susceptibility of individuals to different combinations of driver mutations (95, 97), and v) design personalized therapies and treatments (94, 97, 98). Related to the potential use of organoids to evaluate GBM treatment response, there are some ongoing promising trials in other cancers that demonstrated their powerful and utility. The use of this approach is especially important in cancer treatment due to the inherent resistance of cancer cells and the different response to the treatment among patients. Vlachogiannis et al. demonstrated that cultured cancer-derived organoids from patients with gastrointestinal metastatic cancers treated with a broad set of anticancer agents could retrospectively predict response with an 88% positive predictive value and 100% negative predictive value using a generalized cell viability assay (101). Several clinical studies showed the feasibility of testing patient-derived tumor organoids for evaluation of sensitivity to chemotherapy (102, 103) and radiation. Ooft et al., demonstrated that patient-derived tumor organoids predicted response of the biopsied lesion in more than 80% of metastatic colorectal cancer patients treated with irinotecan-based therapies without misclassifying patients who would have benefited from treatment (102). Therefore, organoid models are not only very useful for studies of essential tumor biology, but also they are suitable for preclinical investigations, such us drug screening and analysis of antitumor effects accompanied by a rapid and safety test in the same system (104).

### 3D Bioprinting in Glioblastoma

Three dimensional biological constructions (3D bioprinting) represent a new and promising method of study not only in GBM but also in other types of diseases (**Figure 2E**). Layers of biomaterials are deposited generating an extracellular matrix, which contains live cells of different types organized into a cell network resembling the real tumor in a very faithful way (105). Particularly, most studies on 3D bioprinting of GBM have been used to study the role of glioblastoma-associated macrophages (GAMs), which are key cells in tumor progression, angiogenesis and also in invasiveness (165–167). The advantage of 3D bioprinting resides in the possibility to replicate the architecture of tissues being crucial to test drugs effectively and also, fulfilling the principle of 3R of animal experimentation (106).

This technology allows the creation of a “mini-brain” in the form of 3D bioprinting model and although it is not a real representation owing to the lack of stem cells, the model includes a combination of different types of cells that are able to interact with each other. The technique is based on bioprinting a brain model using mouse macrophages which is then filled with mouse glioblastoma cells (GL261). The resulting model is adequate to test chemotherapeutic agents as well as macrophage modulating drugs (106). Other authors such as Hermida et al. used another bioprinting method to produce multilineage GBM models (109) and reveal that it is an advantageous method to test drugs and to perform cell signaling analysis using fluorescence-bound protein kinase reporters. Other authors use biomaterials such as hyaluronic acid (HA)-based hydrogels and also, synthetic polymers such as polyethylene-glycol (107, 108).

Furthermore, Tang et al. analyzed 3D printed GBM macrophages in combination with GSC alone or in combination with astrocytes and NPC. They identified molecular characteristics of GSC and evidenced the use of this type of model as an important approach for drug sensibility scanning, the study of invasion, immunologic interactions, and cellular crosstalk (110).

Advances in 3D bioprinting have represented a new vision in the discovery of effective drugs for this type of pathologies, but there are also disadvantages that must be taken into consideration. One is the materials required to print, which may not be physiological molecules. Also, it is necessary to improve the current resolution of printing in order to introduce vasculature (106). Comparing with other methods raised above, the 3D bioprinting is the most adequate when it comes to analyzing the interactions between cells and also to understand the microenvironment created within the tumor (**Figure 2E** and **Table 1**).

## Future Directions

Despite the greatest advances made within the field, GBM is still a highly malignant tumor, resistant to the currently available treatments. Research in the field of GBM should not only be guided towards understanding the behavior of the tumors, but also towards the finding of a new and effective medication. Therefore, the development of experimental models in the study of GBM should be focused on those that facilitate the discovery of new and more potent therapeutic options. In this context, attention needs to be paid to therapies directed to exploiting the potential of the immune system (70, 71). Future experimental models in the study of GBM would need to allow the study of the crosstalk between GBM and the components of the immune system in order to facilitate the development of immune based therapies. In addition, techniques such as GBM organoids that allow the understanding of the individual behavior of each tumor as well as the screening of the available pharmacological options for each individual tumor would be those preferably developed in the short future.

## Conclusions

As time has passed since the first discoveries and advances in the study of GBM using cultures of cells (patient-derived cells and GBM cells lines), and mouse models (xenograft, genetically engineered and viral vector-mediated transduction models) new techniques are now defining the future of GBM studies, which will probably be characterized by the use of individual organoids combined with single cell sequencing of genetic alterations to understand the processes involved in GBM origin and development. Also, the combination of different techniques, such as organotypic cultures and organoids, with 3D bioprinting could lead to an improvement in the study of cell interactions in GBM. Using all these latest scientific advances, targeted therapies can be tested and designed specifically for each patient resulting in a better prognosis and shedding light into the mechanisms controlling this devastating disease.

## Author Contributions

CC, PN-A, and NG-D contributed to the conception and design of the review. RG-O wrote the first draft of the review and created **Figures 1** and **2**. NG-D organized the table information. SD-G, JA-M, LC, CV, RP-D, CC, PN-A, and NG-D wrote sections of the review. All authors contributed to the article and approved the submitted version.

## Funding

This work was supported by the Spanish Ministerio de Ciencia, Innovación y Universidades (Grant Numbers RTI2018–099908-B-C21) and co-financed by the 2014-2020 ERDF Operational Programme and by the Department of Economy, Knowledge, Business and University of the Regional Government of Andalusia. Project reference: FEDER-UCA18-106647. This work has been co-financed by the Integrated Territorial Investment Operational Programme of the European Commission and by the Department of Department of Health and Families (Consejería de Salud y Familias) of the Regional Government of Andalusia. Project reference: ITI-0042-2019 ITI CÁDIZ 2019.

## Conflict of Interest

The authors declare that the research was conducted in the absence of any commercial or financial relationships that could be construed as a potential conflict of interest.
